# Supplementary material for: Olfactory markers for depression: Differences between bipolar and unipolar patients
Source: PLoS One. 2020 Aug 13;15(8):e0237565. doi: 10.1371/journal.pone.0237565 (PMC7426149; doi:10.1371/journal.pone.0237565)
Supplement: S1 Table — Two-by-two comparisons between groups using Tukey test. α = 0.05 (DB: depressed bipolar patients. n = 33; EB: euthymic bipolar patients. n = 30; DU: depressed unipolar patients. n = 33; EU: euthymic unipolar patients. n = 31 and HC: healthy controls. n = 49). d: Cohen’s effect size. (DOCX) [file pone.0237565.s001.docx]

**S1 Table. Demographic and clinical characteristics of patients: Depressive episodes:** two-by-two comparisons between groups using Tukey test. α=0.05 (DB: depressed bipolar patients. n=33; EB: euthymic bipolar patients. n=30; DU: depressed unipolar patients. n=33; EU: euthymic unipolar patients. n=31 and HC: healthy controls. n=49). d: Cohen’s effect size.

| **Group vs Group** | **Group means (SD)** | | **p-value** | **d** |
| --- | --- | --- | --- | --- |
| EU vs DB | 2 (1.5) | 8.8 (10.9) | < 0.0001 | 0.87 |
| EU vs EB | 2 (1.5) | 4.6 (4.2) | 0.382 | 0.82 |
| EU vs DU | 2 (1.5) | 3.5 (3.7) | 0.768 | 0.53 |
| DU vs DB | 3.5 (3.7) | 8.8 (10.9) | 0.004 | 0.65 |
| DU vs EB | 3.5 (3.7) | 4.6 (4.2) | 0.910 | 0.28 |
| EB vs DB | 4.6 (4.2) | 8.8 (10.9) | 0.038 | 0.51 |
