# Supplementary material for: Olfactory markers for depression: Differences between bipolar and unipolar patients
Source: PLoS One. 2020 Aug 13;15(8):e0237565. doi: 10.1371/journal.pone.0237565 (PMC7426149; doi:10.1371/journal.pone.0237565)
Supplement: S2 Table — Two-by-two comparisons between groups using Tukey test. α = 0.05 (DB: depressed bipolar patients. n = 33; EB: euthymic bipolar patients. n = 30; DU: depressed unipolar patients. n = 33; EU: euthymic unipolar patients. n = 31 and HC: healthy controls. n = 49). d: Cohen’s effect size. (DOCX) [file pone.0237565.s002.docx]

**S2 Table. Demographic and clinical characteristics of patients: Hospital admissions:** two-by-two comparisons between groups using Tukey test. α=0.05 (DB: depressed bipolar patients. n=33; EB: euthymic bipolar patients. n=30; DU: depressed unipolar patients. n=33; EU: euthymic unipolar patients. n=31 and HC: healthy controls. n=49). d: Cohen’s effect size.

| **Group vs Group** | **Group means (SD)** | | **p-value** | **d** |
| --- | --- | --- | --- | --- |
| EU vs DB | 0.1 (0.4) | 4.5 (6.1) | < 0.0001 | 1.02 |
| EU vs DU | 0.1 (0.4) | 1.7 (1.9) | 0.290 | 1.17 |
| EU vs EB | 0.1 (0.4) | 1.5 (2.7) | 0.409 | 0.73 |
| EB vs DB | 1.5 (2.7) | 4.5 (6.1) | 0.005 | 0.64 |
| EB vs DU | 1.5 (2.7) | 1.7 (1.9) | 0.998 | 0.09 |
| DU vs DB | 1.7 (1.9) | 4.5 (6.1) | 0.007 | 0.62 |
