# Supplementary material for: Olfactory markers for depression: Differences between bipolar and unipolar patients
Source: PLoS One. 2020 Aug 13;15(8):e0237565. doi: 10.1371/journal.pone.0237565 (PMC7426149; doi:10.1371/journal.pone.0237565)
Supplement: S3 Table — Two-by-two comparisons between groups using Tukey test. α = 0.05 (DB: depressed bipolar patients. n = 33; EB: euthymic bipolar patients. n = 30; DU: depressed unipolar patients. n = 33; EU: euthymic unipolar patients. n = 31 and HC: healthy controls. n = 49). d: Cohen’s effect size. (DOCX) [file pone.0237565.s003.docx]

**S3 Table. Demographic and clinical characteristics of patients: Montgomery Åsberg Depression Rating Scale (MADRS):** two-by-two comparisons between groups using Tukey test. α=0.05 (DB: depressed bipolar patients. n=33; EB: euthymic bipolar patients. n=30; DU: depressed unipolar patients. n=33; EU: euthymic unipolar patients. n=31 and HC: healthy controls. n=49). d: Cohen’s effect size.

| **Group vs Group** | **Group means (SD)** | | **p-value** | **d** |
| --- | --- | --- | --- | --- |
| HC vs DB | 1.6 (2.9) | 41.3 (8.3) | < 0.0001 | 6.39 |
| HC vs DU | 1.6 (2.9) | 39.3 (8.4) | < 0.0001 | 6.00 |
| HC vs EU | 1.6 (2.9) | 2.2 (2.2) | 0.988 | 0.23 |
| HC vs EB | 1.6 (2.9) | 2.0 (2.0) | 0.997 | 0.16 |
| EB vs DB | 2.0 (2.0) | 41.3 (8.3) | < 0.0001 | 6.51 |
| EB vs DU | 2.0 (2.0) | 39.3 (8.4) | < 0.0001 | 6.11 |
| EB vs EU | 2.0 (2.0) | 2.2 (2.2) | 1.000 | 0.10 |
| EU vs DB | 2.2 (2.2) | 41.3 (8.3) | < 0.0001 | 6.44 |
| EU vs DU | 2.2 (2.2) | 39.3 (8.4) | < 0.0001 | 6.04 |
| DU vs DB | 39.3 (8.4) | 41.3 (8.3) | 0.589 | 0.24 |
