# Supplementary material for: Olfactory markers for depression: Differences between bipolar and unipolar patients
Source: PLoS One. 2020 Aug 13;15(8):e0237565. doi: 10.1371/journal.pone.0237565 (PMC7426149; doi:10.1371/journal.pone.0237565)
Supplement: S4 Table — Two-by-two comparisons between groups using Tukey test. α = 0.05 (DB: depressed bipolar patients. n = 33; EB: euthymic bipolar patients. n = 30; DU: depressed unipolar patients. n = 33; EU: euthymic unipolar patients. n = 31 and HC: healthy controls. n = 49). d: Cohen’s effect size. (DOCX) [file pone.0237565.s004.docx]

**S4 Table. Demographic and clinical characteristics of patients: Young Mania Rating Scale (YMRS):** two-by-two comparisons between groups using Tukey test. α=0.05 (DB: depressed bipolar patients. n=33; EB: euthymic bipolar patients. n=30; DU: depressed unipolar patients. n=33; EU: euthymic unipolar patients. n=31 and HC: healthy controls. n=49). d: Cohen’s effect size.

| **Group vs Group** | **Group means (SD)** | | **p-value** | **d** |
| --- | --- | --- | --- | --- |
| EU vs EB | 0.1 (0.4) | 0.8 (1.4) | 0.095 | 0.68 |
| EU vs DB | 0.1 (0.4) | 0.6 (1.6) | 0.296 | 0.43 |
| EU vs DU | 0.1 (0.4) | 0.5 (0.9) | 0.574 | 0.57 |
| EU vs HC | 0.1 (0.4) | 0.1 (0.5) | 1.000 | 0.45 |
| HC vs EB | 0.1 (0.5) | 0.8 (1.4) | 0.052 | 0.67 |
| HC vs DB | 0.1 (0.5) | 0.6 (1.6) | 0.207 | 0.42 |
| HC vs DU | 0.1 (0.5) | 0.5 (0.9) | 0.482 | 0.55 |
| DU vs EB | 0.5 (0.9) | 0.8 (1.4) | 0.822 | 0.25 |
| DU vs DB | 0.5 (0.9) | 0.6 (1.6) | 0.990 | 0.08 |
| DB vs EB | 0.6 (1.6) | 0.8 (1.4) | 0.973 | 0.13 |
