# Supplementary material for: Olfactory markers for depression: Differences between bipolar and unipolar patients
Source: PLoS One. 2020 Aug 13;15(8):e0237565. doi: 10.1371/journal.pone.0237565 (PMC7426149; doi:10.1371/journal.pone.0237565)
Supplement: S5 Table — Two-by-two comparisons between groups using Tukey test. α = 0.05 (DB: depressed bipolar patients. n = 33; EB: euthymic bipolar patients. n = 30; DU: depressed unipolar patients. n = 33; EU: euthymic unipolar patients. n = 31 and HC: healthy controls. n = 49). d: Cohen’s effect size. (DOCX) [file pone.0237565.s005.docx]

**S5 Table. Demographic and clinical characteristics of patients: Physical Anhedonia:** two-by-two comparisons between groups using Tukey test. α=0.05 (DB: depressed bipolar patients. n=33; EB: euthymic bipolar patients. n=30; DU: depressed unipolar patients. n=33; EU: euthymic unipolar patients. n=31 and HC: healthy controls. n=49). d: Cohen’s effect size.

| **Group vs Group** | **Group means (SD)** | | **p-value** | **d** |
| --- | --- | --- | --- | --- |
| HC vs DU | 14.0 (7.1) | 25.0 (9.0) | < 0.0001 | 1.36 |
| HC vs DB | 14.0 (7.1) | 24.0 (8.7) | < 0.0001 | 1.26 |
| HC vs EU | 14.0 (7.1) | 17.0 (9.0) | 0.621 | 0.37 |
| HC vs EB | 14.0 (7.1) | 16.4 (9.5) | 0.678 | 0.29 |
| EB vs DU | 16.4 (9.5) | 25.0 (9.0) | 0.002 | 0.93 |
| EB vs DB | 16.4 (9.5) | 24.0 (8.7) | 0.005 | 0.83 |
| EB vs EU | 16.4 (9.5) | 17.0 (9.0) | 1.000 | 0.06 |
| EU vs DU | 17.0 (9.0) | 25.0 (9.0) | 0.002 | 0.89 |
| EU vs DB | 17.0 (9.0) | 24.0 (8.7) | 0.005 | 0.79 |
| DB vs DU | 24.0 (8.7) | 25.0 (9.0) | 0.999 | 0.11 |
