# Supplementary material for: Olfactory markers for depression: Differences between bipolar and unipolar patients
Source: PLoS One. 2020 Aug 13;15(8):e0237565. doi: 10.1371/journal.pone.0237565 (PMC7426149; doi:10.1371/journal.pone.0237565)
Supplement: S6 Table — Two-by-two comparisons between groups using Tukey test. α = 0.05 (DB: depressed bipolar patients. n = 33; EB: euthymic bipolar patients. n = 30; DU: depressed unipolar patients. n = 33; EU: euthymic unipolar patients. n = 31 and HC: healthy controls. n = 49). d: Cohen’s effect size. (DOCX) [file pone.0237565.s006.docx]

**S6 Table. Demographic and clinical characteristics of patients: Social Anhedonia:** two-by-two comparisons between groups using Tukey test. α=0.05 (DB: depressed bipolar patients. n=33; EB: euthymic bipolar patients. n=30; DU: depressed unipolar patients. n=33; EU: euthymic unipolar patients. n=31 and HC: healthy controls. n=49). d: Cohen’s effect size.

| **Group vs Group** | **Group means (SD)** | | **p-value** | **d** |
| --- | --- | --- | --- | --- |
| HC vs DU | 9.9 (5.7) | 19.6 (6.0) | < 0.0001 | 1.66 |
| HC vs DB | 9.9 (5.7) | 18.8 (6.2) | < 0.0001 | 1.50 |
| HC vs EB | 9.9 (5.7) | 14.4 (7.8) | 0.019 | 0.66 |
| HC vs EU | 9.9 (5.7) | 13.0 (5.6) | 0.193 | 0.55 |
| EU vs DU | 13.0 (5.6) | 19.6 (6.0) | 0.000 | 1.14 |
| EU vs DB | 13.0 (5.6) | 18.8 (6.2) | 0.002 | 0.98 |
| EU vs EB | 13.0 (5.6) | 14.4 (7.8) | 0.912 | 0.21 |
| EB vs DU | 14.4 (7.8) | 19.6 (6.0) | 0.010 | 0.75 |
| EB vs DB | 14.4 (7.8) | 18.8 (6.2) | 0.041 | 0.62 |
| DB vs DU | 18.8 (6.2) | 19.6 (6.0) | 0.990 | 0.13 |
