# Supplementary material for: Olfactory markers for depression: Differences between bipolar and unipolar patients
Source: PLoS One. 2020 Aug 13;15(8):e0237565. doi: 10.1371/journal.pone.0237565 (PMC7426149; doi:10.1371/journal.pone.0237565)
Supplement: S7 Table — Two-by-two comparisons between groups using Tukey test. α = 0.05 (DB: depressed bipolar patients. n = 33; EB: euthymic bipolar patients. n = 30; DU: depressed unipolar patients. n = 33; EU: euthymic unipolar patients. n = 31 and HC: healthy controls. n = 49). d: Cohen’s effect size. (DOCX) [file pone.0237565.s007.docx]

**S7 Table. Demographic and clinical characteristics of patients: STAI - Trait:** two-by-two comparisons between groups using Tukey test. α=0.05 (DB: depressed bipolar patients. n=33; EB: euthymic bipolar patients. n=30; DU: depressed unipolar patients. n=33; EU: euthymic unipolar patients. n=31 and HC: healthy controls. n=49). d: Cohen’s effect size.

| **Group vs Group** | **Group means (SD)** | | **p-value** | **d** |
| --- | --- | --- | --- | --- |
| HC vs DB | 39.8 (9.5) | 60.7 (9.3) | < 0.0001 | 2.22 |
| HC vs DU | 39.8 (9.5) | 55.4 (11.1) | < 0.0001 | 1.51 |
| HC vs EU | 39.8 (9.5) | 44.3 (8.6) | 0.260 | 0.50 |
| HC vs EB | 39.8 (9.5) | 44.3 (10.6) | 0.274 | 0.45 |
| EB vs DB | 44.3 (10.6) | 60.7 (9.3) | < 0.0001 | 1.64 |
| EB vs DU | 44.3 (10.6) | 55.4 (11.1) | 0.000 | 1.02 |
| EB vs EU | 44.3 (10.6) | 44.3 (8.6) | 1.000 | 0 |
| EU vs DB | 44.3 (8.6) | 60.7 (9.3) | < 0.0001 | 1.83 |
| EU vs DU | 44.3 (8.6) | 55.4 (11.1) | < 0.0001 | 1.12 |
| DU vs DB | 55.4 (11.1) | 60.7 (9.3) | 0.198 | 0.52 |
