# Supplementary material for: Olfactory markers for depression: Differences between bipolar and unipolar patients
Source: PLoS One. 2020 Aug 13;15(8):e0237565. doi: 10.1371/journal.pone.0237565 (PMC7426149; doi:10.1371/journal.pone.0237565)
Supplement: S8 Table — Two-by-two comparisons between groups using Tukey test. α = 0.05 (DB: depressed bipolar patients. n = 33; EB: euthymic bipolar patients. n = 30; DU: depressed unipolar patients. n = 33; EU: euthymic unipolar patients. n = 31 and HC: healthy controls. n = 49). d: Cohen’s effect size. (DOCX) [file pone.0237565.s008.docx]

**S8 Table. Demographic and clinical characteristics of patients: STAI - State:** two-by-two comparisons between groups using Tukey test. α=0.05 (DB: depressed bipolar patients. n=33; EB: euthymic bipolar patients. n=30; DU: depressed unipolar patients. n=33; EU: euthymic unipolar patients. n=31 and HC: healthy controls. n=49). d: Cohen’s effect size.

| **Group vs Group** | **Group means (SD)** | | **p-value** | **d** |
| --- | --- | --- | --- | --- |
| EB vs DB | 31.3 (9.0) | 61.1 (14.3) | < 0.0001 | 2.49 |
| EB vs DU | 31.3 (9.0) | 57.8 (15.6) | < 0.0001 | 2.08 |
| EB vs EU | 31.3 (9.0) | 31.7 (9.3) | 1.000 | 0.04 |
| EB vs HC | 31.3 (9.0) | 31.4 (9.0) | 1.000 | 0.01 |
| HC vs DB | 31.4 (9.0) | 61.1 (14.3) | < 0.0001 | 2.49 |
| HC vs DU | 31.4 (9.0) | 57.8 (15.6) | < 0.0001 | 2.07 |
| HC vs EU | 31.4 (9.0) | 31.7 (9.3) | 1.000 | 0.03 |
| EU vs DB | 31.7 (9.3) | 61.1 (14.3) | < 0.0001 | 2.44 |
| EU vs DU | 31.7 (9.3) | 57.8 (15.6) | < 0.0001 | 2.03 |
| DU vs DB | 57.8 (15.6) | 61.1 (14.3) | 0.772 | 0.22 |
