# Supplementary material for: Olfactory markers for depression: Differences between bipolar and unipolar patients
Source: PLoS One. 2020 Aug 13;15(8):e0237565. doi: 10.1371/journal.pone.0237565 (PMC7426149; doi:10.1371/journal.pone.0237565)
Supplement: S9 Table — Two-by-two comparisons between groups using Tukey test. α = 0.05 (DB: depressed bipolar patients. n = 33; EB: euthymic bipolar patients. n = 30; DU: depressed unipolar patients. n = 33; EU: euthymic unipolar patients. n = 31 and HC: healthy controls. n = 49). d: Cohen’s effect size. (DOCX) [file pone.0237565.s009.docx]

**S9 Table. Odors’ identification:** Two-by-two comparisons between groups using Tukey test. α=0.05 (DB: depressed bipolar patients. n=33; EB: euthymic bipolar patients. n=30; DU: depressed unipolar patients. n=33; EU: euthymic unipolar patients. n=31 and HC: healthy controls. n=49). d: Cohen’s effect size.

| **Group vs Group** | **Group means (SD)** | | **p-value** | **d** |
| --- | --- | --- | --- | --- |
| DU vs EU | 9.52 (1.99) | 10.74 (1.59) | 0.017 | 0.68 |
| DU vs HC | 9.52 (1.99) | 10.65 (0.99) | 0.013 | 0.72 |
| DU vs DB | 9.52 (1.99) | 9.94 (1.62) | 0.806 | 0.23 |
| DU vs EB | 9.52 (1.99) | 9.73 (1.72) | 0.982 | 0.11 |
| EB vs EU | 9.73 (1.72) | 10.74 (1.59) | 0.092 | 0.61 |
| EB vs HC | 9.73 (1.72) | 10.65 (0.99) | 0.088 | 0.66 |
| EB vs DB | 9.73 (1.72) | 9.94 (1.62) | 0.985 | 0.13 |
| DB vs EU | 9.94 (1.62) | 10.74 (1.59) | 0.247 | 0.5 |
| DB vs HC | 9.94 (1.62) | 10.65 (0.99) | 0.259 | 0.53 |
| HC vs EU | 10.65 (0.99) | 10.74 (1.59) | 0.999 | 0.07 |
