# Supplementary material for: Olfactory markers for depression: Differences between bipolar and unipolar patients
Source: PLoS One. 2020 Aug 13;15(8):e0237565. doi: 10.1371/journal.pone.0237565 (PMC7426149; doi:10.1371/journal.pone.0237565)
Supplement: S10 Table — Two-by-two comparisons between groups using Tukey test. α = 0.05 (DB: depressed bipolar patients. n = 33; EB: euthymic bipolar patients. n = 30; DU: depressed unipolar patients. n = 33; EU: euthymic unipolar patients. n = 31 and HC: healthy controls. n = 49). d: Cohen’s effect size. (DOCX) [file pone.0237565.s010.docx]

**S10 Table. Odor threshold:** Two-by-two comparisons between groups using Tukey test. α=0.05 (DB: depressed bipolar patients. n=33; EB: euthymic bipolar patients. n=30; DU: depressed unipolar patients. n=33; EU: euthymic unipolar patients. n=31 and HC: healthy controls. n=49). d: Cohen’s effect size.

| **Group vs Group** | **Group means (SD)** | | **p-value** | **d** |
| --- | --- | --- | --- | --- |
| DU vs HC | 9.02 (3.64) | 11.02 (3.2) | 0.044 | 0.58 |
| DU vs EU | 9.02 (3.64) | 10.32 (2.24) | 0.470 | 0.43 |
| DU vs EB | 9.02 (3.64) | 9.78 (3.5) | 0.874 | 0.21 |
| DU vs DB | 9.02 (3.64) | 9.26 (2.98) | 0.998 | 0.07 |
| DB vs HC | 9.26 (2.98) | 11.02 (3.2) | 0.102 | 0.57 |
| DB vs EU | 9.26 (2.98) | 10.32 (2.24) | 0.662 | 0.40 |
| DB vs EB | 9.26 (2.98) | 9.78 (3.5) | 0.965 | 0.16 |
| EB vs HC | 9.78 (3.5) | 11.02 (3.2) | 0.448 | 0.37 |
| EB vs EU | 9.78 (3.5) | 10.32 (2.24) | 0.963 | 0.18 |
| EU vs HC | 10.32 (2.24) | 11.02 (3.2) | 0.874 | 0.25 |
