# Supplementary material for: Olfactory markers for depression: Differences between bipolar and unipolar patients
Source: PLoS One. 2020 Aug 13;15(8):e0237565. doi: 10.1371/journal.pone.0237565 (PMC7426149; doi:10.1371/journal.pone.0237565)
Supplement: S11 Table — Two-by-two comparisons between groups: Tukey test. α = 0.05 (DB: depressed bipolar patients. n = 33; EB: euthymic bipolar patients. n = 30; DU: depressed unipolar patients. n = 33; EU: euthymic unipolar patients. n = 31 and HC: healthy controls. n = 49). d: Cohen’s effect size. (DOCX) [file pone.0237565.s011.docx]

**S11 Table. Hedonic scores for the positive (POS) and the negative (NEG) odors:** Two-by-two comparisons between groups: Tukey test. α=0.05 (DB: depressed bipolar patients. n=33; EB: euthymic bipolar patients. n=30; DU: depressed unipolar patients. n=33; EU: euthymic unipolar patients. n=31 and HC: healthy controls. n=49). d: Cohen’s effect size.

| **Group vs Group according to odor** | **Group means (SD)** | | **p-value** | **d** |
| --- | --- | --- | --- | --- |
| DB*NEG odor vs EU*NEG odor | 2.41 (2.02) | 6.95 (1.69) | 0.081 | 2.44 |
| DB*NEG odor vs DU*NEG odor | 2.41 (2.02) | 3.31 (1.98) | 0.439 | 0.45 |
| DB*NEG odor vs EB*NEG odor | 2.41 (2.02) | 3.07 (1.55) | 0.854 | 0.37 |
| DB*NEG odor vs HC*NEG odor | 2.41 (2.02) | 2.93 (1.49) | 0.926 | 0.29 |
| HC*NEG odor vs EU*NEG odor | 2.93 (1.49) | 6.95 (1.69) | 0.676 | 2.52 |
| HC*NEG odor vs DU*NEG odor | 2.93 (1.49) | 3.31 (1.98) | 0.991 | 0.22 |
| HC*NEG odor vs EB*NEG odor | 2.93 (1.49) | 3.07 (1.55) | 1.000 | 0.09 |
| EB*NEG odor vs EU*NEG odor | 3.07 (1.55) | 6.95 (1.69) | 0.938 | 2.39 |
| DU*NEG odor vs EB*NEG odor | 3.31 (1.98) | 3.07 (1.55) | 1.000 | 0.13 |
| DU*NEG odor vs EU*NEG odor | 3.31 (1.98) | 6.95 (1.69) | 0.998 | 1.98 |
| DB*POS odor vs HC*POS odor | 5.02 (1.3) | 7.33 (1.18) | < 0.0001 | 1.86 |
| DB*POS odor vs EU*POS odor | 5.02 (1.3) | 6.95 (1.69) | < 0.0001 | 1.28 |
| DB*POS odor vs EB*POS odor | 5.02 (1.3) | 6.78 (1.71) | < 0.0001 | 1.16 |
| DB*POS odor vs DU*POS odor | 5.02 (1.3) | 6.09 (1.61) | 0.000 | 0.73 |
| DU*POS odor vs HC*POS odor | 6.09 (1.61) | 7.33 (1.18) | < 0.0001 | 0.88 |
| DU*POS odor vs EU*POS odor | 6.09 (1.61) | 6.95 (1.69) | 0.011 | 0.52 |
| DU*POS odor vs EB*POS odor | 6.09 (1.61) | 6.78 (1.71) | 0.115 | 0.42 |
| EB*POS odor vs HC*POS odor | 6.78 (1.71) | 7.33 (1.18) | 0.267 | 0.37 |
| EB*POS odor vs EU*POS odor | 6.78 (1.71) | 6.95 (1.69) | 0.999 | 0.10 |
| EU*POS odor vs HC*POS odor | 6.95 (1.69) | 7.33 (1.18) | 0.775 | 0.26 |
| DB*NEG odor vs HC*POS odor | 2.41 (2.02) | 7.33 (1.18) | < 0.0001 | 2.97 |
| DB*NEG odor vs EU*POS odor | 2.41 (2.02) | 6.95 (1.69) | < 0.0001 | 0.00 |
| DB*NEG odor vs EB*POS odor | 2.41 (2.02) | 6.78 (1.71) | < 0.0001 | 0.04 |
| DB*NEG odor vs DU*POS odor | 2.41 (2.02) | 6.09 (1.61) | < 0.0001 | 0.03 |
| DB*NEG odor vs DB*POS odor | 2.41 (2.02) | 5.02 (1.3) | < 0.0001 | 0.28 |
| HC*NEG odor vs HC*POS odor | 2.93 (1.49) | 7.33 (1.18) | < 0.0001 | 3.27 |
| HC*NEG odor vs EU*POS odor | 2.93 (1.49) | 6.95 (1.69) | < 0.0001 | 2.52 |
| HC*NEG odor vs EB*POS odor | 2.93 (1.49) | 6.78 (1.71) | < 0.0001 | 2.40 |
| HC*NEG odor vs DU*POS odor | 2.93 (1.49) | 6.09 (1.61) | < 0.0001 | 0.03 |
| HC*NEG odor vs DB*POS odor | 2.93 (1.49) | 5.02 (1.3) | < 0.0001 | 1.49 |
| EB*NEG odor vs HC*POS odor | 3.07 (1.55) | 7.33 (1.18) | < 0.0001 | 3.09 |
| EB*NEG odor vs EU*POS odor | 3.07 (1.55) | 6.95 (1.69) | < 0.0001 | 2.39 |
| EB*NEG odor vs EB*POS odor | 3.07 (1.55) | 6.78 (1.71) | < 0.0001 | 2.27 |
| EB*NEG odor vs DU*POS odor | 3.07 (1.55) | 6.09 (1.61) | < 0.0001 | 1.91 |
| EB*NEG odor vs DB*POS odor | 3.07 (1.55) | 5.02 (1.3) | < 0.0001 | 1.36 |
| DU*NEG odor vs HC*POS odor | 3.31 (1.98) | 7.33 (1.18) | < 0.0001 | 2.47 |
| DU*NEG odor vs EU*POS odor | 3.31 (1.98) | 6.95 (1.69) | < 0.0001 | 1.98 |
| DU*NEG odor vs EB*POS odor | 3.31 (1.98) | 6.78 (1.71) | < 0.0001 | 1.88 |
| DU*NEG odor vs DU*POS odor | 3.31 (1.98) | 6.09 (1.61) | < 0.0001 | 1.54 |
| DU*NEG odor vs DB*POS odor | 3.31 (1.98) | 5.02 (1.3) | < 0.0001 | 1.02 |
| EU*NEG odor vs HC*POS odor | 3.64 (2.00) | 7.33 (1.18) | < 0.0001 | 2.25 |
| EU*NEG odor vs EU*POS odor | 3.64 (2.00) | 6.95 (1.69) | < 0.0001 | 1.79 |
| EU*NEG odor vs EB*POS odor | 3.64 (2.00) | 6.78 (1.71) | < 0.0001 | 1.69 |
| EU*NEG odor vs DU*POS odor | 3.64 (2.00) | 6.09 (1.61) | < 0.0001 | 1.35 |
| EU*NEG odor vs DB*POS odor | 3.64 (2.00) | 5.02 (1.3) | 0.002 | 0.82 |
