# Supplementary material for: Olfactory markers for depression: Differences between bipolar and unipolar patients
Source: PLoS One. 2020 Aug 13;15(8):e0237565. doi: 10.1371/journal.pone.0237565 (PMC7426149; doi:10.1371/journal.pone.0237565)
Supplement: S12 Table — Correspond to the maximum sum of sensitivity and specificity, when all cutoff values of the variable are considering for each group of subjects. *: Accuracy is the Proportion Correctly Classified: (number of True Positives + number of True Negative) / (number of True Positives + number of False Positives + number of False Negatives + number of True Negative). CI: confidence interval. (DOCX) [file pone.0237565.s012.docx]

**S12 Table. Optimal cutoff values:** Correspond to the maximum sum of sensitivity and specificity, when all cutoff values of the variable are considering for each group of subjects.

*: Accuracy is the Proportion Correctly Classified : (number of True Positives + number of True Negative) / (number of True Positives + number of False Positives + number of False Negatives + number of True Negative).

CI: confidence interval

| **Olfactory test** | **Optimal Cutoff value** | **Sensitivity**  **(95% CI)** | **Specificity**  **(95% CI)** | **Sensitivity + specificity** |
| --- | --- | --- | --- | --- |
| **Olfactory threshold** |  |  |  |  |
| DB | 11.25 | 0.73 (0.56-0.85) | 0.59 (0.45-0.72) | 1.32 |
| DU | 11.25 | 0.73 (0.56-0.85) | 0.59 (0.45-0.72) | 1.32 |
| EB | 11.25 | 0.60 (0.42-0.75) | 0.59 (0.45-0.72) | 1.19 |
| EU | 12.75 | 0.90 (0.74-0.97) | 0.33 (0.21-0.47) | 1.23 |
| **Identification** |  |  |  |  |
| DB | 11.00 | 0.64 (0.47-0.78) | 0.59 (0.45-0.72) | 1.23 |
| DU | 10.00 | 0.39 (0.25-0.56) | 0.88 (0.75-0.95) | 1.27 |
| EB | 9.00 | 0.30 (0.17-0.48) | 0.98 (0.88-1.00) | 1.28 |
| EU | 10.00 | 0.16 (0.07-0.33) | 0.88 (0.75-0.95) | 1.04 |
| **Pleasantness (POS)** |  |  |  |  |
| DB | 6.00 | 0.60 (0.54-0.65) | 0.77 (0.73-0.81) | 1.37 |
| DU | 6.00 | 0.48 (0.42-0.53) | 0.77 (0.73-0.81) | 1.25 |
| EB | 5.00 | 0.22(0.17-0.27) | 0.93 (0.91-0.95) | 1.15 |
| EU | 6.00 | 0.34 (0.29-0.40) | 0.77 (0.73-0.81) | 1.11 |
| **Pleasantness (NEG)** |  |  |  |  |
| DB | 2.00 | 0.61 (0.51-0.70) | 0.63 (0.55-0.71) | 1.24 |
| DU | 2.00 | 0.41 (0.32-0.51) | 0.63 (0.55-0.71) | 1.05 |
| EB | 0.50 | 0.37 (0.27-0.47) | 0.75 (0.67-0.81) | 1.11 |
| EU | 0.00 | 0.00 (0.00-0.05) | 1.00 (0.97-1.00) | 1.00 |
| **Familiarity** |  |  |  |  |
| DB | 5.50 | 0.46 (0.41-0.51) | 0.80 (0.76-0.83) | 1.25 |
| DU | 6.00 | 0.42 (0.38-0.47) | 0.78 (0.75-0.81) | 1.20 |
| EB | 6.00 | 0.37 (0.32-0.42) | 0.78 (0.75-0.81) | 1.15 |
| EU | 5.00 | 0.12 (0.09-0.16) | 0.90 (0.87-0.92) | 1.02 |
| **Intensity** |  |  |  |  |
| DB | 5.50 | 0.39 (0.35-0.44) | 0.75 (0.71-0.78) | 1.14 |
| DU | 6.00 | 0.37 (0.33-0.42) | 0.71 (0.67-0.74) | 1.08 |
| EB | 5.00 | 0.12 (0.09-0.16) | 0.93 (0.90-0.95) | 1.05 |
| EU | 6.00 | 0.30 (0.25-0.34) | 0.71 (0.67-0.74) | 1.00 |
| **Emotion** |  |  |  |  |
| DB | 4.00 | 0.33 (0.29-0.38) | 0.86 (0.83-0.89) | 1.19 |
| DU | 5.00 | 0.33 (0.28-0.37) | 0.82 (0.79-0.85) | 1.15 |
| EB | 0.50 | 0.17 (0.13-0.21) | 0.96 (0.94-0.97) | 1.13 |
| EU | 5.00 | 0.27 (0.23-0.32) | 0.82 (0.79-0.85) | 1.09 |
