# Supplementary material for: Olfactory markers for depression: Differences between bipolar and unipolar patients
Source: PLoS One. 2020 Aug 13;15(8):e0237565. doi: 10.1371/journal.pone.0237565 (PMC7426149; doi:10.1371/journal.pone.0237565)
Supplement: S13 Table — Pretest performed on two groups of subjects with similar demographic characteristics to those of the individuals participating in the main experiment. The demographic and psychometric characteristics of the participants are presented in the table below. The results of the odors’ identification test (Sniffin’ sticks identification test–Screening 12 Test) of the two groups of subjects (subjects with depressive symptoms: DS and Healthy controls: HC) are also reported. (DOCX) [file pone.0237565.s013.docx]

**S13 Table. Pre-test:** Pretest performed on two groups of subjects with similar demographic characteristics to those of the individuals participating in the main experiment. The demographic and psychometric characteristics of the participants are presented in the table below. The results of the odors’ identification test (Sniffin’ sticks identification test – Screening 12 Test) of the two groups of subjects (subjects with depressive symptoms: DS and Healthy controls: HC) are also reported.

|  | ***Subjects with depressive symptoms (DS)***  ***(n=36)*** | ***Healthy controls***  ***(HC)***  ***(n=36)*** |
| --- | --- | --- |
| **Female/male ratio** | 27/9 | 26/10 |
| **Mean age, years (SD)** | 34.83 (13.42) | 35.11 (12.42) |
| **Smoker/no smoker ratio** | 9/27 | 7/29 |
| **Educational level, mean (SD)** | 2.94 (0.23) | 2.83 (0.38) |
| **QIDS, mean score (SD)** | 11.19 (2.94) | 2.22 (1.07) |
| **QIDS Range** | 8-17 | 0-4 |
| **Odors’ identification mean score (SD)** | 9.75 (1.18) | 10.78 (1.02) |

*QIDS: Quick Inventory of Depressive Symptomatology; SD: standard deviation.*
