# Supplementary figures and images for: Olfactory markers for depression: Differences between bipolar and unipolar patients
Source: PLoS One. 2020 Aug 13;15(8):e0237565. doi: 10.1371/journal.pone.0237565 (PMC7426149; doi:10.1371/journal.pone.0237565)

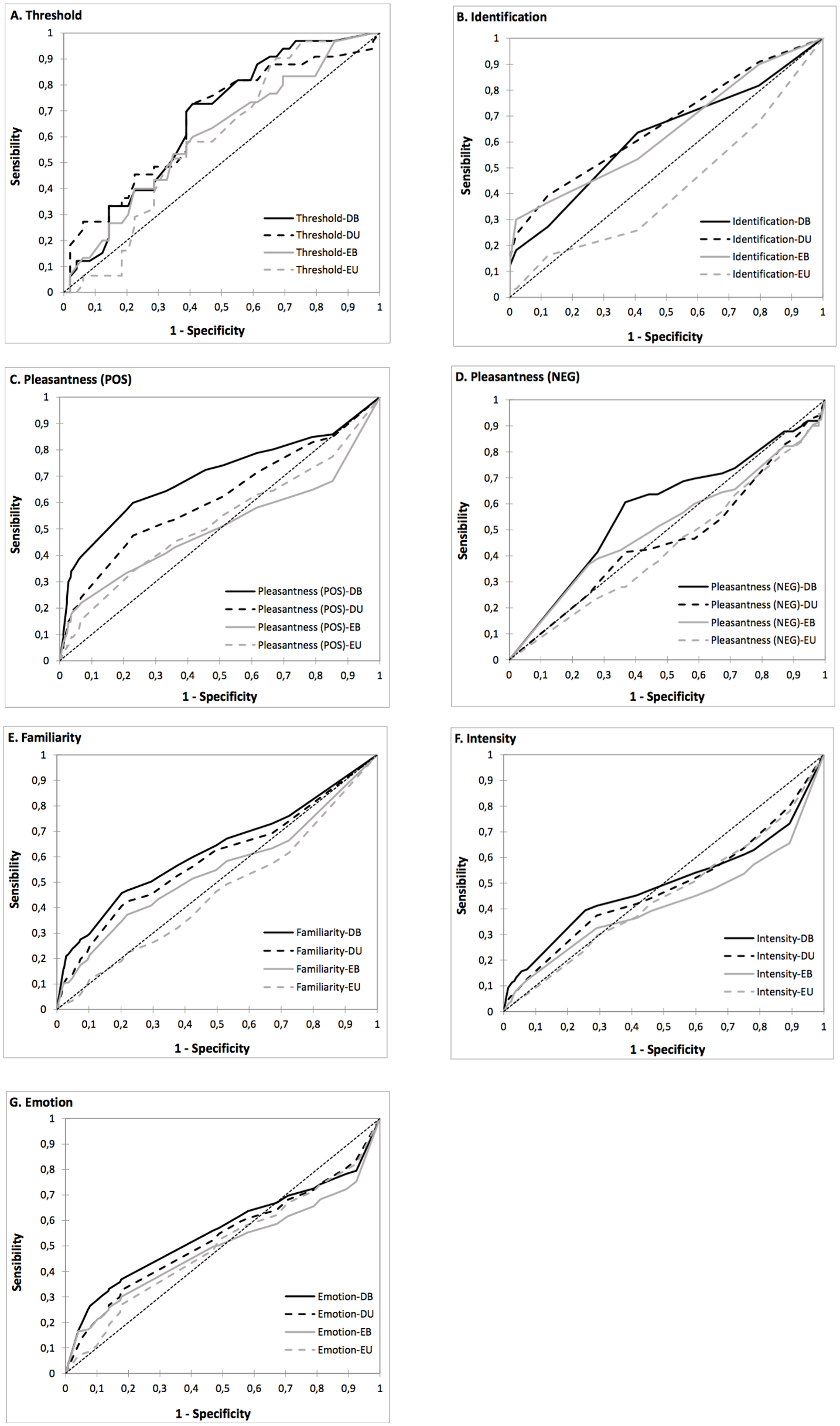

Supplement: S1 Fig — Presentation of the ROC curves for each patients’ group (DB: depressed bipolar patients; EB: euthymic bipolar patients; DU: depressed unipolar patients; EU: euthymic unipolar patients and HC: healthy controls) concerning all olfactory tests. A receiver operating characteristic (ROC) curve plots the true positive rate (sensitivity) against the false positive rate (1 –specificity) for all possible cutoff values. a. Olfactory threshold. b. Identification. c. Pleasantness (POS). d. Pleasantness (NEG). e. Familiarity. f. Intensity. g. Emotion. (TIFF) [file pone.0237565.s014.tiff]
