# Supplementary material for: Olfactory markers for depression: Differences between bipolar and unipolar patients
Source: PLoS One. 2020 Aug 13;15(8):e0237565. doi: 10.1371/journal.pone.0237565 (PMC7426149; doi:10.1371/journal.pone.0237565)
Supplement: Attachment — Submitted filename: Response to Reviewers .docx [file pone.0237565.s015.docx]

PONE-D-19-27567

**Olfactory markers for depression: differences between bipolar and unipolar patients**

Response to Reviewers

Dear Editor,

On behalf of the authors, I would like to thank you and the reviewers for their constructive comments. Our study is one among very few comparing olfactory functions in different groups of subjects with depression (unipolar/bipolar/remission/symptomatic). In this revision, we have taken in account all the comments of the reviewers in order to improve the quality of the manuscript. We have revised the introduction, removed unnecessary information in order to focus on the subject of our study. We have also stated explicitly the hypotheses as requested by the reviewer. We have also added a section related to neuroimaging studies and their links to olfaction and depression as suggested by the reviewer. Finally, we have adapted to manuscript to the requirements of your esteemed journal. Please find below the answers to the specific points raised by the reviewers.

We hope that the changes made will satisfy the editor and the reviewers and allow the publication of this study.

Yours Faithfully,

François Kazour

**Response to the Reviewers

Reviewer #1:**

1. “Introduction is too long, and it seems to missing points.”

**Response: Sections of the introduction that are not related directly to the subject were either deleted or reduced following the suggestion of the reviewer. New information was added to Introduction and discussion (clear hypotheses, neuroimaging and anatomical findings) (according to suggestions of reviewers) in order to have a better and comprehensive approach of the subject.**

2. “Discussion section may be divided into sections.”

**Response: Discussion was divided into sections as suggested by the reviewer**

3. “Each results were interesting, however, it was hard to understand the connection between those results. For example, threshold level in DU was significantly lower than HC, on the one hand, identification of DU was significantly lower than EU and HC. These results can understand DU was clearly different from HC but it was not clear DB and EB. It may be discuss such detail difference in discussion with references of the past researches.”

**Response: Indeed, as the reviewer noticed major olfactory differences were found between unipolar and bipolar depressed subjects:**

**-Olfactory threshold and global identification of smells were affected only in unipolar patients and not in bipolar ones.**

**- Regarding the identification of Positive (Pleasant Odors), both types of depression were affected, but only unipolar subjects would recover their deficit after remission**

**These olfactory differences between unipolar and bipolar subjects were discussed more clearly in the discussion section as suggested by the reviewer (page 21)**

4. “Recent neuroimaging studies showed olfactory brain activations and volume changes related to level of olfactory ability in psychiatric disorders. It needs to discuss the results obtained this study and possibility of past results of the neuroimaging studies.”

**Response: As suggested by the reviewer, we used past and recent references to discuss how abnormalities in neuroimaging studies can explain the olfactory deficits seen in depression (p 20)**

5. “Why there was no difference between groups for percentage of correct response of unpleasant odor and hedonic scores? But pleasant odor had.”

**Response: As suggested by the reviewer, our results show a significant difference between groups in olfactory identification and hedonic rating of pleasant (positive) odors only. This result is due to olfactory anhedonia for pleasant stimuli seen in depressed subjects [Atanasova et al., 2010]. As for unpleasant (negative) odors, the presence in our test of only 3 odors (compared to 9 positive odors), may have prevented the appearance of signifant difference between groups. A future use of olfactory tests with more unpleasant odors may show more significant differences between groups. (This clarification was added to the Discussion page 20).**

**Reviewer #2:**

1. “In the introduction, the authors were able to describe their primary and secondary objectives clearly though the authors could have stated their primary and secondary hypotheses explicitly.”

**Response: As suggested by the reviewer, the primary and secondary hypotheses were stated clearly at the end of the introduction section (page 5).**

2. “I could not see any rationale for the number of participants, in terms of sample size calculation or power calculations.”

**Response: The sample size calculations were performed based on our preliminary data for olfactory identification and in order to have a number of participants for a statistical power of 0.8. allowing the detection of an effect size eta²=0.06 (i.e. d=0.5) at a significance threshold of 0.05. This information was added to the manuscript on page 8.**

3. “With regards to the clinical assessment, the authors used the following tools:
1. The Mini International Neuropsychiatric Interview (MINI 5.0.0)
2. Montgomery-Åsberg Depression Rating Scale (MADRS)
3. Young Mania Rating Scale (YMRS)
4. State-Trait Anxiety Inventory (STAI)
5. Chapman physical and social anhedonia questionnaire
The authors did not report the validity of the use of those tests in the Lebanese culture, especially if participants are not native English speakers or if they speak English at all. The authors need to report if the versions of the 5 tools used in the clinical assessment were conducted in English or Arabic. If the authors used Arabic translations, then they need to cite the authors of the Arabic translation of those tools.”

**Response: In this study we used the Arabic validated versions of the MINI (Kadri et al., 2005), the STAI (Hallit et al., 2019) and the MADRS (Hallit et al., 2019). These references were added to the manuscript. As for the YMRS, we used an Arabic translation of this scale since couldn’t find a validated version. However, this scale was used to rule out a manic/hypomanic episodes that were already ruled out by the MINI. As for the Chapman Anhedonia Questionnaire, we did an Arabic translation and a back translation of this questionnaire due to the absence of a validated version. The results obtained in this study were gathered for the validation of the Arabic version of this questionnaire that is still in process. This lack of validation is a limitation of our study and was added to the “Limitations” section of the manuscript.**

4. “With regards to the statistical tests used, the authors decided to use one-way analysis of variance (one-way ANOVA) with Tukey posthoc test when comparisons between groups were carried out for quantitative variables. While the use of parametric ANOVA might be suitable for parametric tests, such as age, other variables, such as educational level, total identification score and threshold scores might not conform to the normal distribution probability model. Hence, the use of Kruskal–Wallis one-way analysis of variance might be more suitable. The authors might want to use Dunn's post hoc tests to examine the difference in each pair of groups.”

**Response: The normal distribution of the data was not always validated (Kolmogorov-Smirnov test). Despite this, the parametric tests were used. Indeed, based on our sample size (n ≥ 30 per group), conditions for applying the central limit theorem was met, allowing us using one-way ANOVA even when normality may not be ensured for all samples (when central limit theorem applied, sampling distributions follow a normal distribution) (This information was added to the manuscript on page 8).**
