# Supplementary material for: Olfactory markers for depression: Differences between bipolar and unipolar patients
Source: PLoS One. 2020 Aug 13;15(8):e0237565. doi: 10.1371/journal.pone.0237565 (PMC7426149; doi:10.1371/journal.pone.0237565)
Supplement: Attachment — Submitted filename: RESPONSE TO REVIEWERS.docx [file pone.0237565.s016.docx]

Dear Editor,

On behalf of the authors, I would like to thank you, as well as the reviewers for the effort you’ve been giving to improve the quality of our manuscript through your constructive comments and remarks. Please find below the responses to Reviewer #2 concerns. I hope that our responses and the modifications we added to the manuscript will provide an adequate answer to their comments.

Yours truly,

**RESPONSES**

Reviewer #2: I am pleased to see that the authors have responded adequately to points 1 and 3.

The authors' response to points number 2 and 4 have not, however, addressed the issues raised satisfactorily.

So, for point number 2 on the calculation of the sample size, while authors have reported “statistical power of 0.8. allowing the detection of an effect size eta2=0.06 (i.e. d=0.5) at a significance threshold of 0.05. “, they did not report the parameter they used to calculate the effect size between the different groups. Did the author use the Olfactory threshold or Olfactory identification-related-parameters to calculate the effect-size? More detailed data about the findings of the pilot need to be included.

**RESPONSE: We would like to thank the reviewer for their comments. Concerning the comment on the calculation of the sample size, we calculated the statistical power (with XLSTAT) by using means and standard deviations of a previous Olfactory identification task. We performed a pretest on 2 groups of subjects with similar demographic characteristics similar to those of the individuals participating in the main experiment (these results have been added to the supplementary data file: Table S.13). In this pretest we obtained an effect size Cohen's d = 0.9339 (for t-test), which is comparable of a eta² = 0.179 (for ANOVA) (Cohen , 1988).**

**For the experiment, we therefore estimated our sample size calculation from a more conservative effect size eta² = 0.06, which corresponds to an effect of intermediate magnitude according to Cohen (1988) and requiring to recruit more participants. Accordingly, the estimation of the sample size for an ANOVA was performed to be able to detect an effect with alpha = 0.05, a power of 0.8 and for an effect size of eta² = 0.06. Hence, the sample size necessary to obtain a power of 0.8 and an effect size of eta² = 0.06 at alpha = 0.05 is 192 participants (if we had kept the less conservative eta² = 0.179 from the pre-test with the same alpha and power, the calculation would have indicated that 60 participants may be enough)In our study, we therefore aimed to recruit more than 192 particpants, and we indeed were able to have 215 participants. However, our criteria made us that we only had 176 subjects have been included in the study.Nevertheless, this number was still much higher than 60. Moreover, with a sample size of 176 we should be able to detect an effect size of 0.065 (with alpha = 0.05 and a power of 0.8, see below), still very sensitive and a more precise effect size than eta² = 0.179.**

| **Parameters** | **Data input** |
| --- | --- |
| Nb of groups : | 5 |
| partiel Eta² : | 0,065 |
| **Parameters** | **Results** |
| Power | 0,8 |
| Alpha | 0,05 |
| Effect size (f) | 0,26366402 |
| Sample size | **176** |
| Power (obtained) | 0,799 |

With regards to the point number 4 concerning the statistical tests used, the authors cited Central Limit Theorem (CLT) and the sample size(n>30) to be sufficient to use parametric tests even if the normality of the distribution of data is not assured. Saying that, CLT requires random sampling from the population of interest. The authors did not report how large was the population they sampled, and if the sampling was done randomly. Hence, without examining the normality of the distribution of the parameters, the authors need to report the details of the sample selection and if this selection was random. The other alternative is to use nonparametric statistics (distribution-free), as mentioned in the previous review.

**RESPONSE: The reviewer comments on the sampling method of our population and the statistical tests used. In our study, we recruited subjects from two hospital clinical settings: all subjects who presented to inpatients and outpatients clinics with depressive symptoms were approached and then either included or excluded from this study. This method of selection was random even if it did not include a true randomization of all the subjects presenting with depressive symptoms in the general population. This method of sampling is seen in most clinical studies, where strict and true randomization is rarely feasible. Therefore, this leads to a possible selection bias that is a limitation to our study whether we use either parametric or non-parametric tests (Matthews JA, 1981; Scheff SW, 2016). This bias is acknowledged and added to the discussion of the manuscript. In our study and based on the Central Limit Theorem, we considered that the distribution of means approaches normality as sample size is > 30 per group and that sample independence was assumed, and therefore justifying the use of parametric statistics like ANOVA (Kwak & Kim, 2017; ; Scheff SW, 2016). Parametric test like ANOVA gives more powerful results compared to non-parametric Kruskal –Wallis test Finally, our sampling method, despite its limitations, is similar to those used in most clinical studies and allows us to reach results that can only be generalized after taking in consideration the biases mentioned above. We hope that our response provides an adequate answer to the justified concerns of the reviewer regarding the sampling and statistical tests that were used.**

- **Cohen, J. (1988). *Statistical power analysis for the behavioral sciences* (2nd ed). Hillsdale, NJ: Erlbaum Associates. DOI:10.4324/9780203771587**
- **Matthews, J. A. (1981). Further Non-parametric Tests for Independent Samples. In *Quantitative and Statistical Approaches to Geography* (p. 108‑123). DOI:10.1016/B978-0-08-024295-8.50017-4**
- **Scheff, S. W. (2016). *Fundamental Statistical Principles for the Neurobiologist : A Survival Guide* (1 edition). Academic Press. DOI:10.1016/C2015-0-02471-6**
- **Kwak, S. G., & Kim, J. H. (2017). Central limit theorem : The cornerstone of modern statistics. *Korean Journal of Anesthesiology*, 70(2), 144‑156. https://doi.org/10.4097/kjae.2017.70.2.144**
