# Supplementary material for: Olfactory markers for depression: Differences between bipolar and unipolar patients
Source: PLoS One. 2020 Aug 13;15(8):e0237565. doi: 10.1371/journal.pone.0237565 (PMC7426149; doi:10.1371/journal.pone.0237565)
Supplement: Attachment — Submitted filename: REPONSES AU REVIEWER_24_7_20.docx [file pone.0237565.s017.docx]

**PONE-D-19-27567R2
Olfactory markers for depression: differences between bipolar and unipolar patients**

Dear Editor,

Dear Reviewer,

On behalf of the authors, I would like to thank you for your patience and for the constructive comments you brought to our manuscript.

As requested, the following modifications were made:

**Additional Editor Comments (if provided): Please, review punctuation throughout the manuscript. Many thanks.**

- Punctuation was reviewed and necessary corrections were made throughout the manuscript

**Reviewer #2: The authors have addressed the two commends raised in the last review. However, table 1 and 3 need reformatting to fit the page.**

- Tables 1 and 3 were reformatted as requested to fit the page
